# Human Contributions to Safety Data Testbed Flight Simulation Study: Data Methods, Processing, and Quality

**DOI:** 10.1038/s41597-025-05336-7

**Published:** 2025-07-16

**Authors:** Tyler Fettrow, Chad Stephens, Lance Prinzel, Jon Holbrook, Kathryn Ballard, Sepehr Bastami, Michael Stewart, Daniel Kiggins

**Affiliations:** 1 NASA Langley Research Center, Hampton, VA 23666 USA; 2 San Jose State University Research Foundation, San Jose, California 95192 USA

**Keywords:** Human behaviour, Decision

## Abstract

NASA’s System-Wide Safety Project achieved a pivotal milestone through a structured observation simulation experiment aiming to transform safety in aviation. Building on prior initiatives, the study investigates pilot behavior and resilience during challenging scenarios. We studied 24 commercial pilots performing simulated real-world challenges during approach to KCLT such as traffic compression, convective weather, and modulating workload. We acquired standard human factors assessments like NASA-TLX and SART, and also collected a variety of psychophysiological measures such as EEG, ECG, and eye tracking. We employed custom questionnaires and retrospective think-aloud exercises to enable quantifying resilient and safe behavior. Video and audio were also recorded from multiple sources. This paper provides details regarding methodological procedures, data management, and a glimpse at some preliminary analyses. The data and code are publicly available providing a dynamic resource that encourages public contribution to quantification of resilient behavior in commercial airline pilots.

## Background & Summary

NASA Aeronautics Research Mission Directorate System-Wide Safety Project marks a significant milestone with the successful completion of a groundbreaking structured observation simulation experiment. The primary objective of this endeavor was to enhance the way we approach safety within the aviation industry by dramatically expanding our understanding of what constitutes a safety-relevant event. We have methodically curated a comprehensive data set reflecting real-world flight operations under challenging safety-of-flight threatening scenarios. We called the study S ystem-Wide Safety O perations and T echnologies for E nabling R esilient I n-Time A ssurance, or “SOTERIA”. This data set promises to provide insights into pilot behavior and dynamics during standard airline operations. Furthermore, this data set will play a pivotal role in refining our understanding of how pilots produce safety^1^ through their capability for resilient performance, as outlined by Hollnagel *et al*.^2^ and Holbrook *et al*.^3^. In this report, we offer a concise overview of our motivations behind this study. Subsequently, we delve into the methodologies employed during its collection. Finally, we offer insights into the data set itself, shedding light on its potential impact on aviation safety.

The motivations for this study stem from the notion that the majority of the actions taken by flight crews to enable safe sustained operations go unmeasured, while rare human errors are extensively studied. Because we cannot learn from data that are not collected or analyzed, this discrepancy reflects a missed opportunity to learn from the majority of what actually happens^4,5^. Thus, this study focuses on some of the more mundane situations that might occur during routine commercial airline flights such as navigating around convective weather, communication with air traffic control, or managing the energy of the aircraft. Here we have generated a data set that will allow us to answer questions based on what happens, not just what fails. The primary research question we sought to answer was, “how do commercial airline pilots manage routine contingencies during Area Navigation (RNAV) arrivals?”. The present study was designed to identify and capture real-world operational behavior through replication of known actual line operational events that have occurred at Charlotte Douglas International Airport (KCLT) in which observable resilient behavior had been described^6^.

In our study, we sought to engage current commercial pilots in a high-fidelity, motion-based Boeing 737 simulator that adheres to standard airline protocols. We developed scenarios around routine operational challenges such as energy management, weather navigation, traffic pattern integration, and communications with Air Traffic Control (ATC). Throughout these scenarios, we collected psychophysiological metrics in a minimally intrusive manner, including electrocardiography (ECG), electroencephalography (EEG), via a mobile brain imaging unit, and galvanic skin conductance via an activity watch. Every scenario was video and audio recorded. Following each scenario pilots provided subjective evaluations of their performance including workload, teamwork, and perceived resilient performance, among others. The data set provides unprecedented insights into both individual and collective pilot behaviors-insights that remain largely uncharted in typical flight operations. This unique data could revolutionize our understanding of productive safety, shaping new paradigms that value proactive and positive safety.

Our team has disseminated initial findings through conference papers, detailing preliminary analyses of the data^7–9^. These early studies drew connections between eye tracking data and subjective resilience assessments, as well as relating the reported workload and resilience scores to expert evaluations of pilot performance, respectively. In the current paper, we describe the experimental methods, the data organization, and data quality assessment-a component not addressed in previous publications. By doing so, we aim to provide a foundation for future research and potential real-world applications in aviation safety.

## Experimental Methods

### Participants, Setup, and Daily Activities

Twenty-six (24) healthy airline transport pilots (15 men; 9 women, Mean age for the group = 49.1 years) currently employed at a major airline in the United States volunteered for the study (individual demographic information available in data repository^10^). All participants had current Class I aeromedical examinations. Participants were briefed on the study, read, and signed informed consent and Privacy Act statements prior to participating in accordance with requirements from the NASA Institutional Review Board (NASA IRB approval Study eIRB Number: STUDY00000477).

Upon arrival for Day 1 data collection, participants were escorted to the briefing room for informed consent, experiment briefing, and psychophysiological setup (~1 hour). Following the informed consent process, each pilot was outfitted with a combined electroencephalography (EEG) and electrocardiography (ECG) device (B-Alert ABM X10; Advanced Brain Monitoring, CA, USA; https://www.advancedbrainmonitoring.com/products/b-alert-x10). The EEG had 9 electrodes on the scalp, 1 on each mastoid process, and 2 in parallel with the heart’s electrical signal on the chest. To enable optimal EEG signal, and Kustomer Kinetics brand Synapse®conductive electrode cream was applied until the impedance of each EEG electrode was verified to be less than 10 megaohms. The ABMX X10 device sampled both EEG and ECG at 256 Hz. Pilots were also outfitted with a smart watch that measured galvanic skin response, skin temperature, and heart rate (E4; Empatica, MA, USA; https://www.empatica.com/research/e4/). The Empatica device sampled different data at different frequencies; The accelerometer sampled at 32 Hz, the electrodermal activity (EDA) at 4 Hz, the temperature at 1 Hz, and the Inter-beat Intervals (IBI) at 64 Hz. Following the checkout of the psychophysiological systems, each pilot proceeded to the simulator flight deck (NASA Langley Research Center Integrated Flight Deck, VA, USA) and performed an eye tracking calibration procedure (Smarteye Pro DX, MA, USA; https://www.smarteye.se/smart-eye-pro/). The smarteye sampled at 60 Hz. These devices have been used extensively in a variety of research aimed at quantifying and assessing workload and stress, both within NASA and among the general research community^11–18^.

The psychophysiological recording units were time synched via a Network Time Protocol server and triggered for recording through eyesDX Multi-modal Analysis of Psychophysiological and Performance Signals (MAPPS; eyesDX, IA, USA; https://www.eyesdx.co/). Videos of six flight displays and the over the shoulder camera were also part of the MAPPs synching. Video and audio were captured and recorded for all scenarios which enabled fusing the flight displays with an over the shoulder camera. We refer to this video recording as Digital Video Recorder (DVR).

**Fig. 1:**
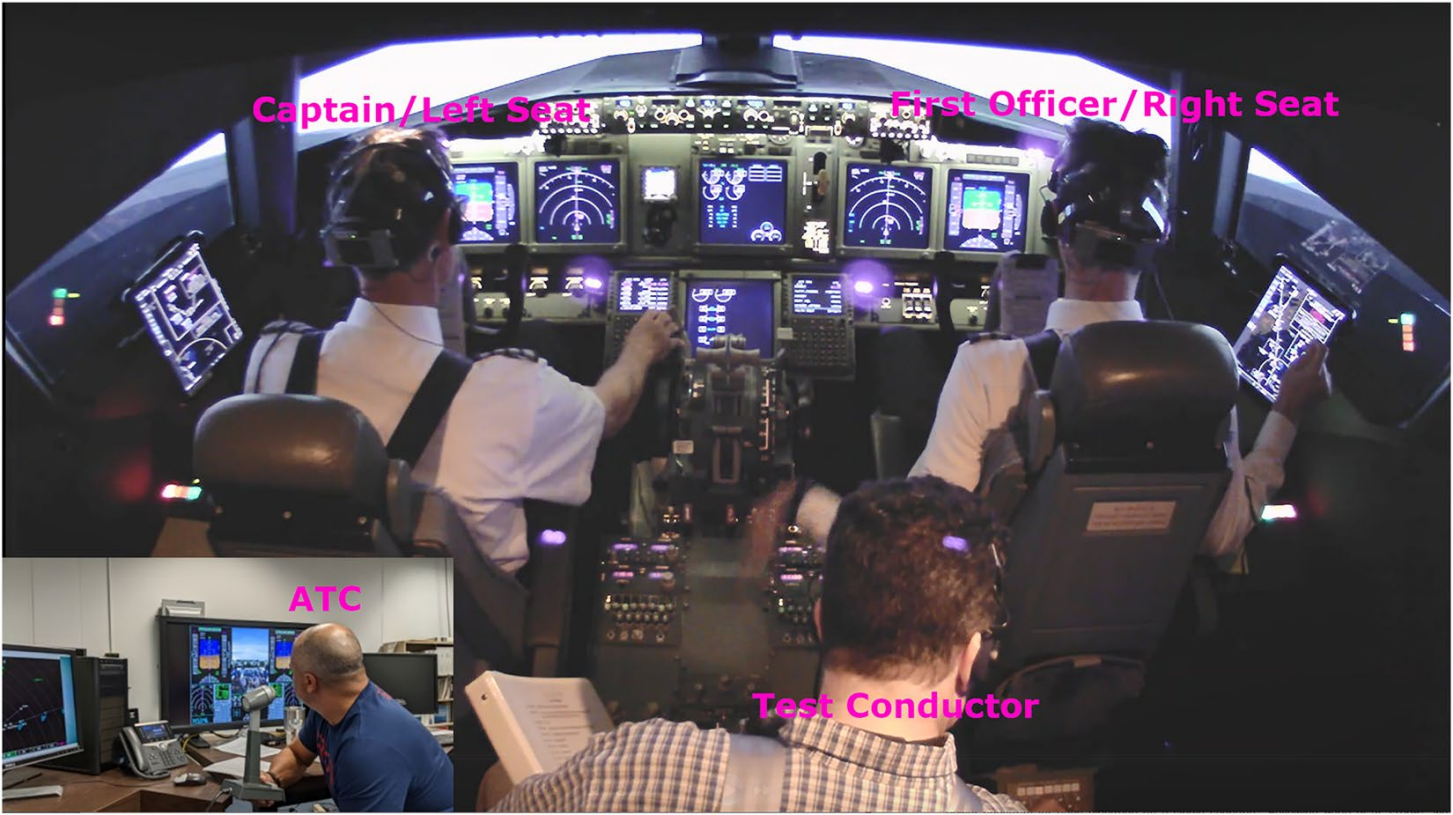
Simulator cockpit setup. The full-motion Integrated Flight Deck operated on a hydraulic 6-Degrees of Freedom platform replicating a Boeing 737-800. The jumpseat behind the pilots allowed the test conductor to easily exchange survey documents with the crew in between scenarios without standing up (safety hazard while platform in operation). The air traffic controller (in the simulation control room) had standard situational awareness of the scenarios, with an additional live-stream of the cockpit.

Once all equipment was verified operational, the pilots then began a familiarization run. This scenario allowed the pilots to get comfortable with the simulator controls, while allowing for questions to be answered by the test conductor in the jumpseat. Figure 1 shows the positioning of the aircrew with respect to the test conductor, as well as the live air traffic controller in a separate room. There was also a live “other pilot” actor to emulate radio chatter during the scenario (not shown). We refer to the captain as left seat and the first officer as right seat throughout the paper and in the data repository^10^.

After each scenario, the test conductor administered multiple surveys including the NASA TLX, Situation Awareness Rating Technique (SART), and the Resilient Performance Self-Assessment (RPSA) developed from American Airlines Learning and Improvement Team (LIT) publications^19,20^. Results of preliminary analysis of the NASA TLX, SART, and RPSA data are described in Stephens *et al*.^8^.

Lunch break was provided around noon for each crew. Depending on how smooth the data collection went that morning, we would break after the third or fourth scenario. During the break for lunch, the pilots were escorted back to the briefing room, and their lunch was delivered to them. Psychophysiological devices were turned off and plugged in to ensure batteries would last the remainder of the day. Occasionally some EEG or ECG electrodes needed to be adjusted to improve site contact. After lunch, pilots were escorted back to the simulator, and devices were turned on and another eye tracking calibration was performed. Time stamps are provided in the trial_settings.txt if interested in distinguishing morning versus afternoon scenarios.

At the end of the first day (after all scenarios were completed), psychophysiological devices were removed from each pilot and additional surveys were administered. The post-study surveys included a pilot comfort survey (in reference to the psychophysiological recording devices) and simulation realism survey^21^. The Ego-Resiliency Scale^22^, the New General Self-efficacy scale^23^, and the “Aviation Resilience Potential Scale” and the “System Resilience Potential” questionnaires (developed from Hoffman and Hancock^24^), were collected prior to the simulation on day 1 of the study. The total duration of Day 1 data collection typically lasted approximately 8 hours.

The pilots returned the day after the simulation for a post-simulation interview which involved multiple activities. First pilots were instructed to write an event report as if they were completing an Aviation Safety Action Program (ASAP) form, for scenario 3 (draft_1). Then the pilots watched a replay of their performance during scenario 3, followed by a rewrite of the event report (draft_2). The event reports are stored as plain text files within the EventReport folder within the data repository. Additionally, the pilots were shown the replay video of their performance in scenario 2 and performed a retrospective think-aloud minute by minute. Crews also assigned a workload rating on a scale of 1-7, for each of those minutes (stored in survey_data.xlsx. Specifically, this involved a researcher playing back the DVR recording for the scenario, playing one minute of video, then pausing for pilot feedback, beginning with the first officer. These sessions were video recorded and are stored in the RetroThinkAloud folder within the data repository.

### Simulation Scenarios

**Fig. 2:**
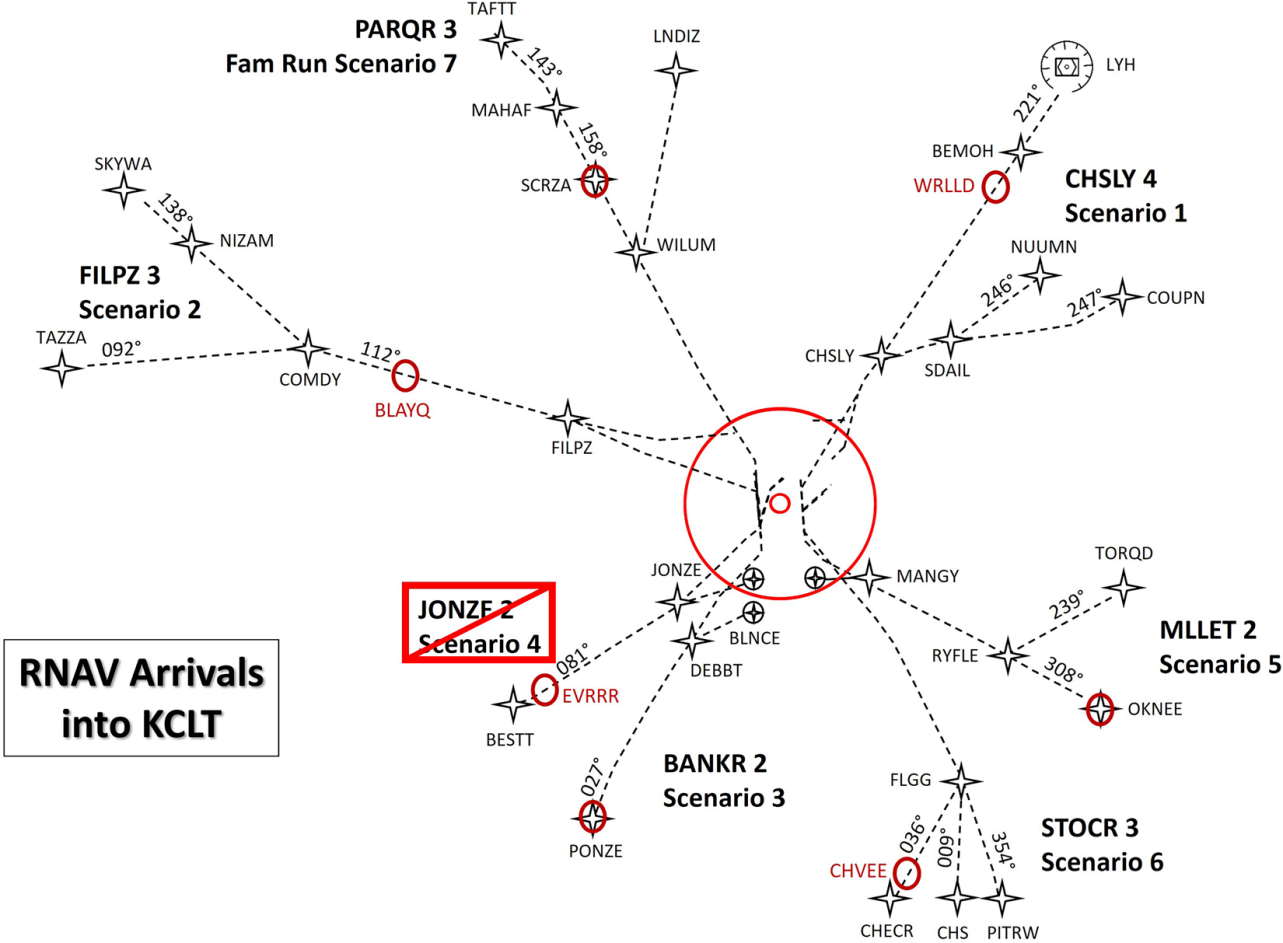
Initial starting conditions around KCLT for each scenario.

All scenarios were based on KCLT area navigation (RNAV) arrivals (see Fig. 2) designed to simulate both foreseeable and unforeseeable disturbances frequently documented in Aviation Safety Reporting System (ASRS), Aviation Safety Action Program (ASAP) reports, airline crew reports, and common high-frequency events at KCLT (e.g., weather and traffic). Although the arrival flight paths were specific to KCLT airspace, the scenarios were designed to enable generalization across fleets and airspace. The arrival operations at KCLT are sufficiently complex to generalize to other major hub airports in the United States and globally^25,26^. Each scenario replicates documented instances of resilient pilot performance in response to these disturbances. These scenarios include two events in which flight crews can display behaviors like anticipation, monitoring, response, and learning.

Each scenario began after top-of-descent, with the aircraft positioned on the RNAV arrival track, maintaining appropriate attitude and airspeed. Pilots receive a detailed scenario synopsis before its commencement, including time for an arrival briefing and flight management system (FMS) entries, similar to real-world operations. Dispatch paperwork was provided for each scenario, and pilots used their company-supplied tablets for enhanced realism. The first officer started every scenario as the pilot flying.

In essence, these scenarios and the simulation environment aim for high-fidelity recreation of commercial line operation arrivals at KCLT, covering event categories such as energy management, traffic compression, convective weather, unanticipated tailwinds, auto-flight issues, icing conditions, system cautions, wake encounters during descent, ATC or pilot clearance errors (e.g., hearback/readback errors), and high workload. Table 1 provides a mapping from the arrival route to the scenario number with which the data was saved. The scenario numbers range from 1-7, but Scenario 4 was not included in the collection due to experiment protocol time limitations. The scenarios were completed in a pseudo-randomized order such that a crew did not complete the scenarios in the same order as another, except that each crew began with the familiarization scenario (Scenario 7). The following subsections describe each scenario in detail.

**Table 1:** Scenario information.

Arrival	Data Name
CHSLY FOUR	Scenario 1
FILPZ THREE	Scenario 2
BANKR TWO	Scenario 3
MLLET TWO	Scenario 5
STOCR THREE	Scenario 6
PARQR THREE	Scenario 7

The Arrival columns provides the arrival track name. The Data Name column provides the name of the scenario as it was saved in the raw data and presents in the current data repository. Scenario 4 was not included in the collection due to experiment protocol time limitations.

#### Scenario 1: CHSLY FOUR

Scenario 1 was designed to represent a common issue observed with KCLT operations that involve significant traffic on the CHSLY FOUR arrival in which traffic merges at CHSLY waypoint. Numerous ASRS reports (e.g., 1517588, 1527071, 1444706) document issues for the arrival particularly when ATC issues significant and/or numerous profile changes. Examples include reports where flight crews were told to maintain speed, then speed up, then slow down, then vectored off profile to be given heading to fly from fix, modified again, and given climb for traffic then direct to fix under high temporal demands (e.g., ASRS report 1444706). The arrival has also been known to be problematic for the 737-800 due to the high energy demands and need for high drag devices between CHSLY (with large window of FL220 - 13000 at 250 KT) and NODEW (9000 - 8000 at 230 KT). Therefore, event 1 is the described energy management issue.

Event 2 for Scenario 1 was a Traffic compression and high flows issue. This event represents a common issue for CHSLY FOUR arrival involving being high in large windows at SLPOH (FL210 - 10000) late runway changes (i.e., arrival chart states to EXPECT RWY 23, but RWY 18L given late in scenario) and “direct to” clearances (direct to HEELZ slow to 210 KT cross at 6000 - a hard altitude chart constraint) designed to modulate traffic flows into KCLT approach airspace. The scenario event provides opportunity for flight crews to be proactive and manage energy based on significant cues provided during scenario if anticipated behavior is observed (e.g., traffic ahead provided clearance for runway change to 18L and direct to HEELZ (with hard altitude of 6000) instead of JEPHS (which is at/above 6000).

#### Scenario 2: FILPZ THREE

Scenario 2 was designed around a common event at KCLT in which convective weather (i.e., thunderstorms) present issues for traffic flows and throughput rates, including need for holds and diverts around cells. The scenario event 1 was represented as a moving thunderstorm front north of the FILPZ THREE arrival track (112 degrees) prior to PHAYE (which has a published HOLD). A path attenuation compensation alert (Wx 2100 radar) was displayed in area, along FLIPZ THREE arrival track 1120, where the attenuated region (i.e., radar shadow) was displayed as an amber arc on outer range ring of navigation display. Traffic stream was set-up so that preceding aircraft continue the arrival approach, including penetration of weather presenting as “green” (with “yellow” areas) on the WXR2100 weather radar. The scenario initiated with aircraft at BLAYQ to ensure time to observe traffic ahead, ATC communications, and developing / moving weather front to make decision(s). The scenario was designed to provide ambiguous cues, including dynamics of the weather, such as does preceding aircraft penetrate the weather, request divert, HOLD at PHAYE or FLIPZ, or other behavior (e.g., inter- and intra-flight deck communications).

Event 2 (continued scenario event category: Weather) was designed to observe how flight crew brief a potential concern for approach and landing based on earlier convective activity (event 1) and increased reports (and evidence) for windshear, turbulence, low-level windshear, and gusts (with associated tailwind landing restrictions for 737-800). KCLT has sophisticated Terminal Doppler Weather Radar and reporting capabilities to plausibly provide advisories/alerts (within 15 nm of airport).

#### Scenario 3: BANKR TWO

Scenario 3 was based on ASRS reports involving high tailwinds from west arrivals (e.g., 1518030, 1607833, 1624966). Event 1 involved encountering tailwinds that differ from those entered as predicted in the Flight Management Computer for certain flight levels (and weather forecast provided to flight crews prior to scenario initiation). Based on documented reports, the event replicated the wind direction and vectors of tailwind at altitude on the BANKR TWO arrival. The impact of the unanticipated winds was to affect spacing of the aircraft on the arrival stream requiring monitoring, anticipating, and responses to mitigate the situation (i.e., energy management of aircraft, communications with ATC, etc.).

Scenario 3 event 2 represented a complex and varied issue that falls under the general category, Autoflight issues. Examples of issues with the autoflight system (e.g., FMS failing to initiate a turn) include ASRS reports 1518030, 1607833, 1624966, and 1605019. After crossing DEBBT (FL210 - 12000 at 250 KT), for operations landing NORTH (RWY 36 L/C/R) the BANKR TWO profile consists of turn from 040 to 070 to CONTR (3.0 NM from DEBBT) and OPALS (6.2 NM from DEBBT). During the scenario, the autopilot failed to initiate a turn at DEBBT and continue along track 040 (toward ROBRR) in which the flight crew would have to monitor for and recognize the issue and manually intervene (e.g., through mode control panel) to initiate a turn back to CONTR (at or above 11000) or OPALS (12000 - 10000), which has a 2000’ window, making the situation more demanding. During event 1, ATC (live ATC) provided an altitude and airspeed clearance to preceding aircraft where that simulated pilot chatter incorrectly read back the clearance (i.e., “readback” error). The incorrect readback, could have potential impact on the participant aircraft planned trajectory. Live ATC re-stated the correct clearance to the preceding aircraft within 5 minutes at which time the simulated pilot chatter correctly read back the clearance. Flexibility was afforded to live ATC and text-to-speech capability to provide realism and timeliness to the communications and any responses from the participant flight crew (e.g., query communication to live confederate controller).

#### Scenario 5: MLLET TWO

The event category for scenario 5 event 1 was “wake encounter”. The scenario initiates on the MLLET TWO arrival at 320 KT landing SOUTH with expect RWY 18L (as charted). The scenario involved a traffic stream situation in which lingering wake turbulence was (potentially) encountered by the flight crew. A number of ASRS reports (e.g., 1543900, 1588529, 1457869) document the issue of potential for wake turbulence even when separation standards are maintained. The scenario 5 event 1 was taken from a report in which a 737-800 was on KCLT RNAV arrival with an Airbus (A321) 7.8 NM ahead with other HEAVY aircraft (e.g., A330) also ahead (i.e., 17 NM) with aircraft in the flow slowing (e.g., high drag devices in use). The wind was a quartering tailwind of 40 KT and the own ship path was below the profile path of that traffic ahead. The scenario event provided an opportunity to monitor for and anticipate the potential for wake turbulence based on pilot-ATC communications, what the aircraft ahead were doing and flight path of the traffic, and wind condition. Potential mitigating actions included request to increase spacing, vector off path, increase altitude, and/or request HOLD. The wake encounter was planned to effect a 20 degree roll to the right, followed by 40 degree roll to the left, and loss of 20 KT during flight through the wake turbulence.

Scenario 5 event 2 involved readback and hearback errors (including copy errors). On the MLLET TWO arrival there was an “EXPECT” to cross at 250 KT at hard altitude constraint of either 12000 (north) or 14000 (south) depending on if landing south or north. The event involved the live ATC confederate intentionally providing an incorrect clearance to cross MLLET at 12000 (runway 18L in use) not 14000 (correct altitude). Traffic clearances ahead and behind the own ship was given correct clearance. The FMS profile was correctly shown in the Control/Display Unit including the 14000 constraints at MLLET and RWY 18L entered as runway.

#### Scenario 6: STOCR THREE

Scenario 6 was focused on high workload to see how flight crews manage tasks and explore alternatives to mitigate temporal demands and challenges of clearance compliance. For event 1 ATC gave a clearance to fly direct on an outbound radial from the Charleston Very High Frequency Omni-directional Range. The set-up to fly outbound on a radial is an acceptable clearance but not an easy set-up and entry for the FMS and display on the Navigation Display.

Event 2 involved a “flipping the airport” situation in which the winds changed sufficiently to require change from South (RWY 18L/R) to North flow operations (RWY 36L/R). To increase workload, aircraft was given a direct to GATEE (6000 at 210 KT) for RWY 36L instead of expected HANDO (8000 at 210 KT) for RWY 18L.

#### Scenario 7: PARQR THREE

The PARQR THREE RNAV was used for the familiarization scenario. This scenario was nominal and allowed the pilots to acclimate to the simulator controls, while allowing for questions to be answered by the test conductor in the jumpseat.

## Data Records

MAPPs data were exported as log files for all data files, and mpegs for the video files recorded by MAPPs. The DVR videos were stored as mp4. The retrospective think-aloud audio was recorded and saved on a laptop. The survey data were manually converted into a digital form, .xlsx for the numeric data (i.e., NASA-TLX, self-reported resilience, etc.), and .txt for the hand written responses (Event Report). The files were transferred from the local recording data collection computer to an external drive and then uploaded to NASA’s Box repository for safe keeping. We copied the contents of this repository to an AWS S3 bucket (see Usage Notes for more details) to enable public access^10^. The version of the data uploaded to AWS prior to April 2025 was peer reviewed.

**Fig. 3:**
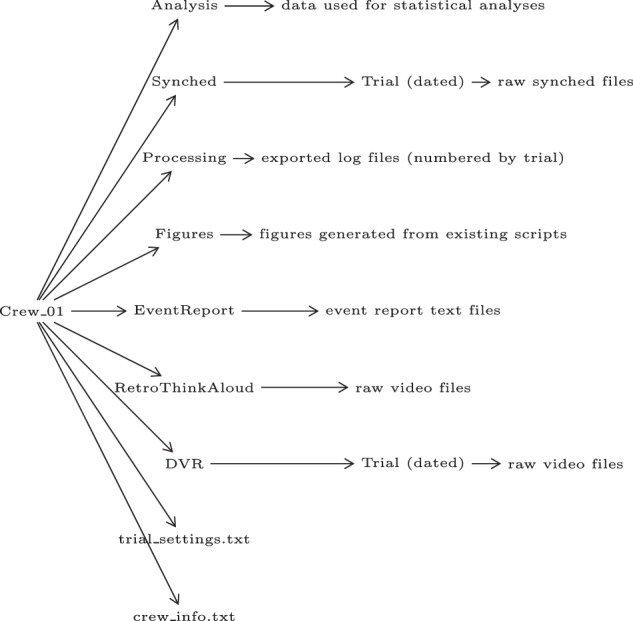
SOTERIA data repository structure. This structure repeats for every crew. Within each crew folder, there are several folders that contain either additional subfolders (in the case of Synched, RetroThinkAloud, and DVR), or a list of files. Additionally, within each crew folder there is a trial_settings.txt and crew_info.txt. The trial settings serves as a mapping between the dates files and the number-by-trial files. The crew info file contains demographic information. The survey data is stored in survey_data.xlsx, and this file sits at the same level as the Crew folders within the data repository, containing Crew and Seat information on each sheet to differentiate the data.

The folder structure is important to discuss. We had a total of twelve crews complete the study. One crew attempted to complete the study but was cut short due to illness, hence in the figures we skip Crew 12 (incomplete). Within the project folder, we have a folder for each crew’s data. Within each Crew folder we have a “Synched” folder which contains the raw data exported from MAPPs. The preprocessed data is exported to .csv data files and saved in a “Processing” folder, which is also in parallel with the Synched folder within the Crew folder. For analyses we create more data-specific data formats, and these data structures are then saved in the “Analysis” folder and the resulting figures are saved in the “Figures” folder, which also reside within the Crew folder in parallel with Synched and Processing folders. See Fig. 3 for a visualization of the folder structure. A pdf (not pictured in Fig. 3) is stored at the top level of the data repository that contains the blank versions of all questionnaires administered throughout the experiment. Additionally, there is a script in the code repository (repoFileDict.py) that will print details about all the files currently in the data repository.

Within the Synched folder there are several sub-folders that are named based on the timestamp of collection end. Due to the randomization of the scenario runs, we needed to create a mapping between the timestamp and scenario number. This was done manually based on data collection notes, and stored in the trial_settings.txt (shown above in file path contents). The txt file contains two columns of information, the first being the timestamp of each sub-folder within the Synched folder, and the second being the scenario that it corresponds to. This mapping is used to automatically find the relevant folders within the Synched folder, and subsequently name the exported data files by scenario.

We performed preprocessing and quality assurance steps to ensure that the data transfers were successful, and also to determine the data loss during the experiment. Due to the size of the videos, there were occasions during the transfer from the external drive to Box that resulted in a full hard drive, and therefore a failed or partial transfer.

First, to ensure that the transfer to Box was successful, we performed an automated comparison of the file sizes between the external drive and the Box repository using a custom Python script that utilized the *os.stat* method and initiated an overwrite if the file sizes were not the same. For the transfer to the AWS S3 bucket, “aws sync” handled the comparison implicitly. Due to occasional network drops, we had to run sync multiple times until the method reported no changes between Box and the Bucket.

We converted the data files from a .log format, to a more universal .csv format, and simultaneously modified the timestamps to start at zero for each run (instead of the GPS time stamp), and reformatted from “H:M:S” to a single float in units of seconds. We then saved the reformatted data in a csv named based on the data type and the contents of the trial_settings.txt.

**Fig. 4:**
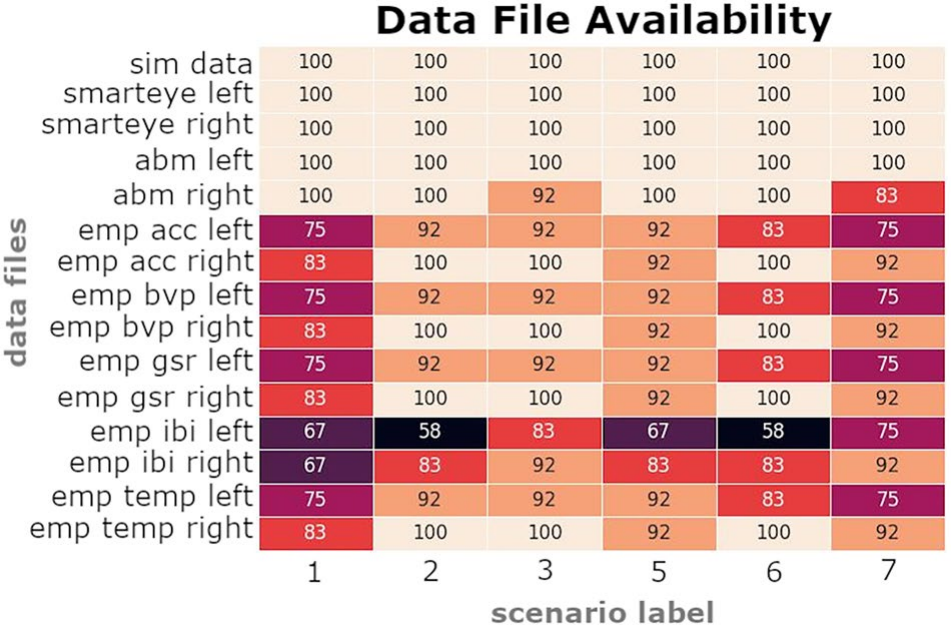
Percentage of total files existing by scenario. The data files listed on the y-axis represent individual data files, but the “abm” file contains both EEG and ECG. Additionally, the “emp” files are all derived from the Empatica device, but each measurement was exported as a separate data file. The “left” and “right” nomenclature refers to the pilot. The sim_data file contains the flight dynamic and pilot input parameters.

There were multiple occasions where the Bluetooth connection of either the smartwatch or the EEG failed during data collection, and did not record a data file. Therefore, after exporting the data, we then checked how much of the exported existed compared to what was expected based on the known file types and the contents of the file_settings.txt (the scenarios that we know we collected). We created binary maps for each subject to enable viewing of how much data was missing. Figure 4 depicts how much data is missing for across all subjects, for each data type and scenario.

The flight simulation and eye tracker (Smarteye) data were always recorded. The flight simulation data is stored in the Synched and Processed folders, within files that begin with ifd_cockpit_scenario*.csv. The flight simulation data was the most critical and therefore was the most obvious data stream that provided evidence that things were working properly. The eye tracking data is hardwired into the simulation setup, therefore the risk of malfunction was much lower compared to other data measurements. The ABM and Empatica devices were transferring data via Bluetooth, and therefore were much less reliable. There were a few instances where the ABM failed to collect data at all, and those are instances are shown in Fig. 4 where “abm right” or “abm left” are not 100%. Occasionally the bluetooth connection dropped, or the bluetooth dongle became loose in its socket preventing data transmission. Similarly, and much more frequently, the Empatica failed to transmit any data, and varied by data type.

The DVR recordings were always recorded. The exception to that is Crew 1 did not contain audio. All DVR recordings were stored as mp4 on the collection computer, transferred to an external hard drive, and finally stored in the data repository within the DVR folder. The retrospective think-aloud was recorded via a smartphone and transferred to an external hard drive. All crews performed this activity on Scenario 2. The retrospective think-aloud recordings were stored as .m4a and transferred to the data repository within the RetroThinkAloud folder.

## Technical Validation

### DVR and Retrospective Think-Aloud Data Quality

Due to the nature of the DVR and Retrospective Think-Aloud data, a technical validation was not necessary. As stated earlier, the DVR was always recorded, with the exception that Crew 1 did not contain audio. The Retrospective Think-Aloud was also collected for every crew. The Retrospective Think-Aloud audio includes the audio of the DVR, but at times it can be difficult to hear. The main objective of this exercise was to acquire pilot perspective about their experience during each minute of scenario played back to them.

### Survey Data Quality

We collected numerous subjective measures from the test subjects, most of which are well vetted and widely used (i.e., NASA-TLX^27^, SART^28^, and rating for simulation fidelity^21^). The Resilience Assessment Grid^29^ has only recently been developed, but is gaining traction in major airline operations^19^. All participants completed every survey form, except for the Crew 2 captain (left seat), who did not complete the Resilience Assessment Grid surveys due to a technical failure.

### Psychophysiology Data Quality

For each data type we ran a “preprocessing” script that served two purposes, first to quality check the data, and second to create data structures for a preliminary analysis. These scripts are publicly available and we encourage their use. See Usage Notes sections for more details. We will describe the data acquired from each psychophysiology device in the following subsections.

#### Eye Tracking (Smarteye)

**Fig. 5:**
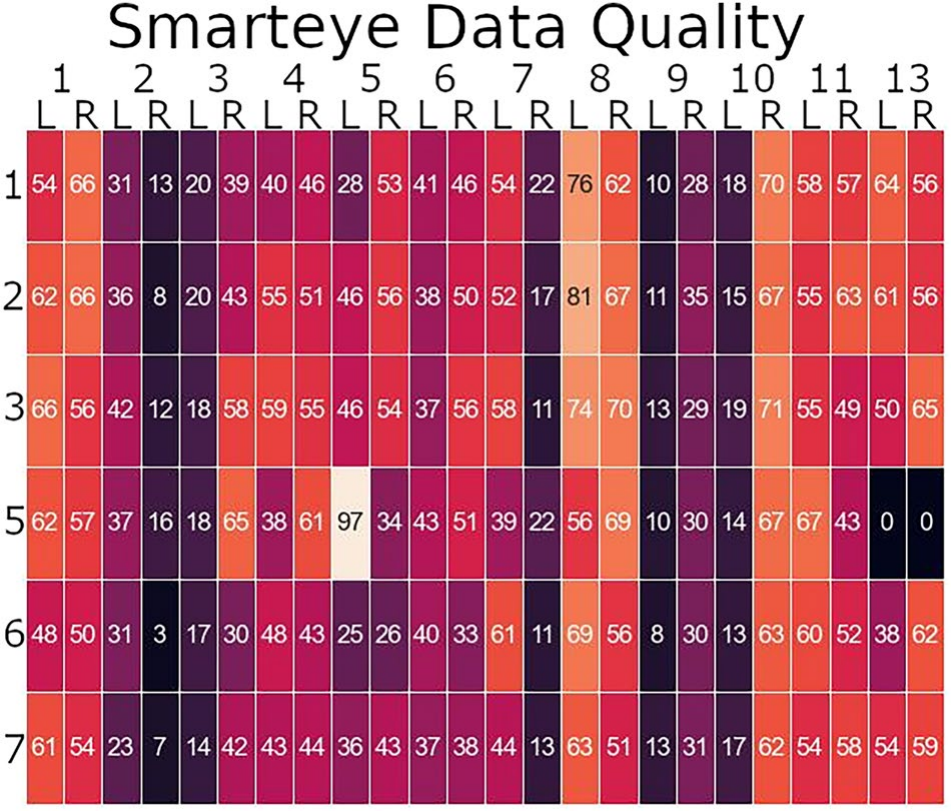
Percentage of smarteye data that is deemed usable. The heatmap is color-coded based on the amount of data that passed our liberal threshold for usable gaze data. The brighter the color, the more data that can be used. The numbers on the x-axis correspond to the crew number, and the L and R correspond to the left and right seat, respectively.

For an initial look at the eye tracking data quality we chose the gaze direction data and pupil diameter as the assessment tool. For gaze direction we converted the unit vector to a plane using standard stereographic mapping^30^. We retained indices where the quality percentage was greater than 60%, and the velocity of the raw gaze vector of respective indices did not exceed 700 degrees/second. Figure 5 shows the percent data available for analysis for each scenario and subject. In general, the percent data available is consistent within a subject across scenarios, except for a couple of anomalies. For example, Crew 5 left seat has a 97% available for scenario 5, and Crew 13 does not have any available for scenario 5. It should be noted that this is a somewhat simplistic method for determining the available data, and there may be methods to improve the data quality through interpolation, filtering, or both.

**Fig. 6:**
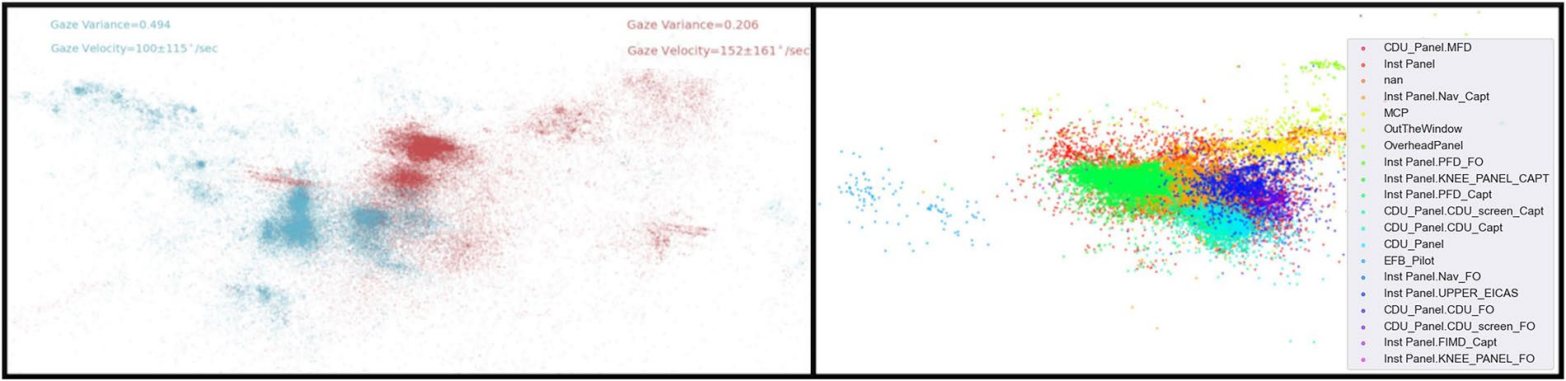
Example of eye tracking gaze data. left figure plots the left pilot (blue), and the right pilot (red) gaze vectors after performing the planar transformation described in the Methods section. Two metrics (gaze variance and velocity) are calculated and displayed in the top corner of the figure for each pilot. The figure on the right displays a single pilot’s planar gaze vector, but now color-coded based on the object that Smarteye software decided they were looking at. Additional post-processing steps can be taken to improve the accuracy of this labeling^14^.

An example of the gaze data for both pilots is shown in Fig. 6. The left figure displays both the left seat (blue) and right seat (red) gaze data that has passed the filtering criteria. The right figure displays an example of a single pilot but the gaze data is colored based on the object with which the fixation occurred (according to the Smarteye post-processing).

#### Electroencephalography (EEG)

**Fig. 7:**
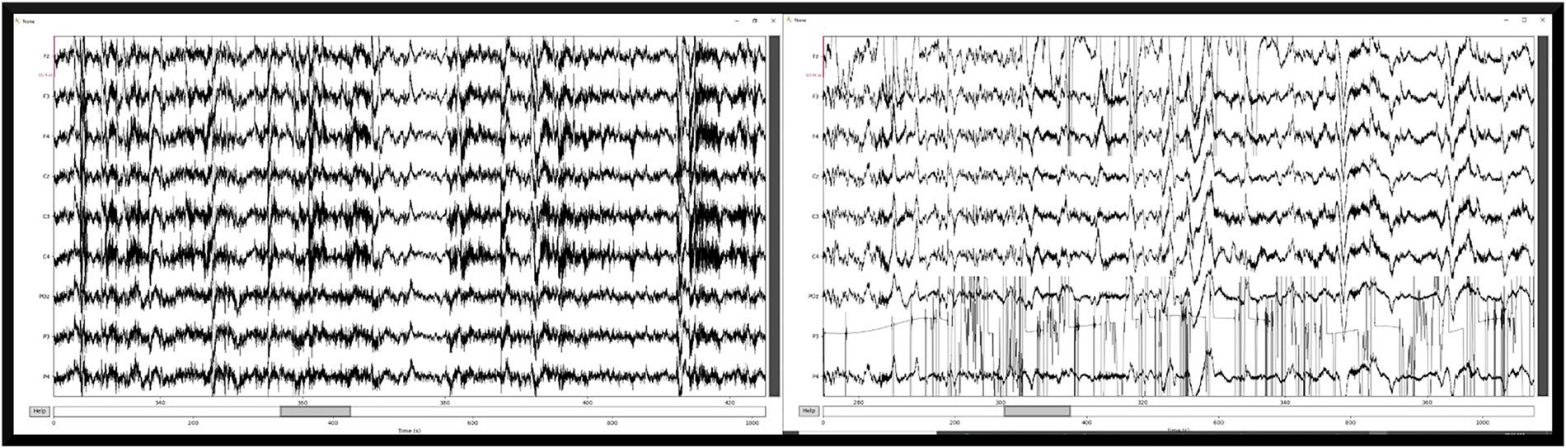
Example of raw EEG data. Raw EEG recordings purpleover 100 seconds. The left figure displays a section of good EEG tracings, whereas the raw EEG recordings on the right display bad tracings for Fz and P3.

The EEG data was the most involved in regards to preprocessing. The device consisted of 9 EEG electrodes, and each electrode has the potential to go awry during a data collection. Therefore we visually assessed each electrode’s raw data to check for consistent noisy data, and generated a spreadsheet based on which electrodes presented the noisy data. We used Python MNE package to filter, label, and plot the data^31,32^. Figure 7 displays an example of one subject’s data for two different scenarios. The figure on the left displays a scenario that had good data for all electrodes, and the right figure displays a scenario where a few electrodes started to introduce noise where Fz and P4 have periodic artifacts and P3 appears to contain completely unusable data. To capture this we created a coded matrix that stores the data quality for each electrode as good, mediocre, or bad. This data is stored in a csv format (eeg_electrode_quality.csv) and is located in the helper folder within the code repository. The visual inspection of EEG data can be arbitrary and imprecise, therefore we encourage new users to deploy their own methods of coding the EEG data quality and contribute back to this dataset.

After filtering and initially looking at the raw data, the EEG preprocessing script calculates the power spectrum for each electrode within each epoch. Looking at how the task load index changes around the scenario events provides one example of how this data could be analyzed. If a user is interested in further assessing and improving the quality of EEG data, see the EEG preprocessing script in the publicly available code repository.

**Fig. 8:**
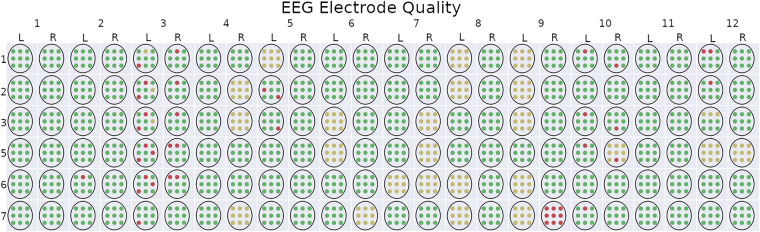
EEG electrode quality map. Each electrode for every crew and scenario is displayed in this figure. The colors correspond to the coding recorded in eeg_electrode_quality.csv. The numbers on the x-axis correspond to the crew number, and the L and R correspond to the left and right seat, respectively. Electrodes that were coded as bad are reflected as red, questionable as yellow, and good as green.

EEG is a rich data signal, therefore there are countless analyses that can be performed on this data. Also, there is room for improving the amount of usable data. Specifically, the way we coded the data (in eeg_electrode_quality.csv) is coded by each electrode for the entirety of each run. So for example, if one electrode looked bad, we coded that bad for the entire run. If an electrode has consistent but periodic movement artifacts, we coded that as “ok”, but for sake of these example figures, we only included electrodes that were coded as “good”. Figure 8 displays the electrode coding map to date, for each subject. Future improvements to coding of the electrodes could be done by identifying the bad sections within the run, as opposed to classifying the entire run as bad.

#### Electrocardiography (ECG)

**Fig. 9:**
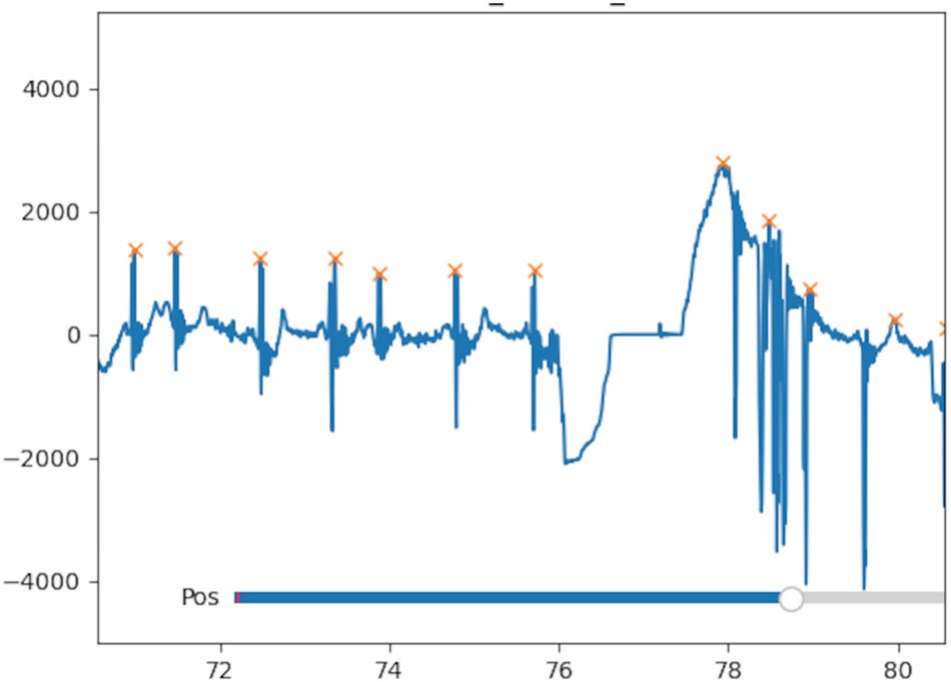
Example of Raw ECG signal. Specifically, an example of the peak fitting algorithm of a section that goes from good to bad (left to right), likely as a result of a movement artifact. Percent of the trial is displayed on the x-axis. In this case, we documented unusable data from 76–79% of the trial in the ecg_quality.xlsx file.

Similar to the EEG, the ECG required manual viewing and coding of the raw signal. However, here we were specifically interested in coding the efficacy of the peak detection algorithm. Therefore, we plotted the ECG signal for every trial and pilot and documented areas where the algorithm was unable to identify a proper heartbeat. We stored the sections that were unusable in the ecg_quality.xlsx file as a percent of the trial. As in Fig. 9, a section of the signal looked inaccurate likely due to a movement artifact, so we coded that area as unusable in the ecg_quality.xlsx file.

**Fig. 10:**
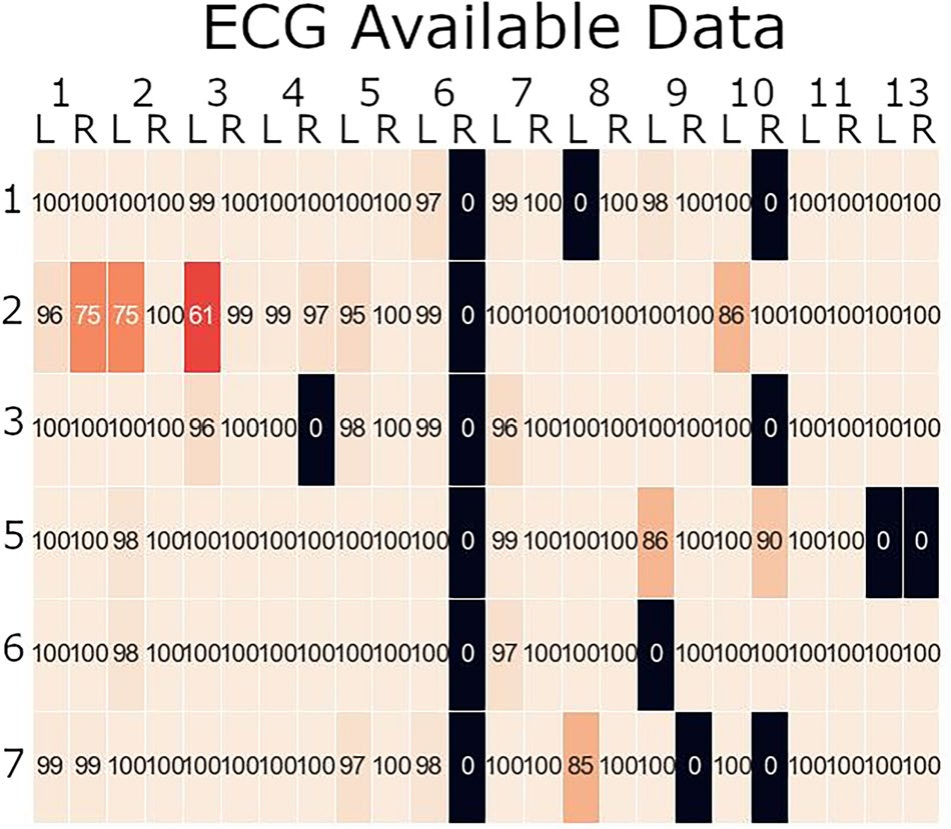
Percentage of ECG data that is deemed usable. The y-axis contains the scenario numbers, while the x-axis contains the pilot information. For each crew there was a left (L) and right (R) pilot. The heat map is color-coded based on the amount of data that we decided was usable after a manual assessment of the peak-detection algorithm. The brighter the color, the more data that can be used.

The ECG preprocessing script reads in the file that stores usable data, and calculates heart rate and heart rate variability in time epochs, excluding the data determined to be unusable (i.e., data was unusable due to signal loss and/or artifacts). This data is then stored in a csv file in the Processing folder for further analysis. The amount of usable ECG data does differ between participants, and in some cases (in a couple occasions the peak detection algorithm did not work well), the amount of usable data can be improved. Figure 10 displays the amount of usable data for each subject and scenario as we determined by our manual assessment of the data described earlier.

#### Wrist worn data sources (Empatica)

The Empatica device resulted in the lowest overall collection rate according to Fig. 4. Moreover, there were numerous occasions where the device would begin collection and writing to file at the start of the scenario, then some time into the scenario would drop connection and therefore lose data for the remainder of the scenario. On occasions where the data was complete, we can gather accelerometery of the wrist, blood volume pulse (BVP), galvanic skin response (GSR), temperature, and inter beat interval (IBI). However, more often than not we would lose one sensor type. Despite the data woes for this particular measuring device, we include the data in the repository for complete transparency and hope that the data that does exists provides values to the users.

## Planned Additions

There are currently several planned additions to the data set that will be uploaded at a future date. First, the most critical is the addition of improved “event” identification. Currently the events are coded based on the timing of a particular call from ATC (i.e., deviate course), or a specific event occurring in the simulation (i.e., wake turbulence). However, it is possible that the ATC call timing differed between crews due to the ability of the crew to foresee the problem, or some other unforeseen circumstance. Regardless, there is a need for a more robust methodology for identifying the stressor events. Since each scenario had video and audio recordings, we plan to have observations completed by The LOSA Collaborative and American Airlines LIT that will provide resilience and workload classifications for each scenario and crew. This will provide expert level classification of events for improved future analyses.

Additionally, we plan on having all the videos transcribed. We have attempted to use language processing software to transcribe, but due to the nuanced nature of air traffic communications, some language was misinterpreted. Therefore we plan on having experts check and correct the automated software results prior to releasing this data publicly.

## Usage Notes

The data is publicly available at the Registry of Open Data on AWS AWS Registry and can be found by searching for “soteria”, or more specifically, “nasa-soteria-data”. The data can be pulled and listed using standard aws cli functions (see AWS CLI section).

We also provide the code that was used to organize, preprocess, and analyze the data presented in this paper. The code is hosted on a publicly available Github repository. The code is entirely written in Python, and will run on any computer so long as Python3 and the appropriate modules are installed. The code repository has a Wiki that describes the steps for setting up the python environment to enable the code’s use, as well how the code is used.

Our primary goal of releasing the data set publicly is to allow the public to help define and classify resilient behavior specifically in aviation operations and in general. The mechanisms for enabling this are the release of a rich, comprehensive data set, along with the code that can be used to organize and preprocess the data. Our hope is that the others will contribute back to both the data and the code repositories as significant improvements are made. Additions to the data set need to be approved by the administrator of the data set, therefore, please reach out to either Tyler Fettrow or Chad Stephens (contact information listed on title page).

## Data Availability

The code is publicly available on Github. As noted in the Usage Notes section, there is a Wiki associated with the code repository that explains in detail the steps required to utilize the code. In short, the code is written in Python and will run on any machine that has Python 3 installed, along with many dependent packages. We also encourage people to contribute to the code base as significant additions are generated, via pull requests.
